# High-precision docking of wheelchair/beds through LIDAR and visual information

**DOI:** 10.3389/fbioe.2024.1446512

**Published:** 2024-09-04

**Authors:** Xiangxiao Lei, Chunxia Tang, Xiaomei Tang

**Affiliations:** ^1^ School of Electronic Information Engineering, Changsha Social Work College, Changsha, China; ^2^ Hunan Victor Petrotech Service Co., Ltd., Changsha, China

**Keywords:** automatic docking, intelligent wheelchair/bed, visual positioning, lidar, IMU

## Abstract

To address the low docking accuracy of existing robotic wheelchair/beds, this study proposes an automatic docking framework integrating light detection and ranging (LIDAR), visual positioning, and laser ranging. First, a mobile chassis was designed for an intelligent wheelchair/bed with independent four-wheel steering. In the remote guidance phase, the simultaneous localization and mapping (SLAM) algorithm was employed to construct an environment map, achieving remote guidance and obstacle avoidance through the integration of LIDAR, inertial measurement unit (IMU), and an improved A* algorithm. In the mid-range pose determination and positioning phase, the IMU module and vision system on the wheelchair/bed collected coordinate and path information marked by quick response (QR) code labels to adjust the relative pose between the wheelchair/bed and bed frame. Finally, in the short-range precise docking phase, laser triangulation ranging was utilized to achieve precise automatic docking between the wheelchair/bed and the bed frame. The results of multiple experiments show that the proposed method significantly improves the docking accuracy of the intelligent wheelchair/bed.

## 1 Introduction

To improve the quality of life of people with lower limb motor dysfunction and reduce the labor intensity of nursing staff, the development of assistive robots for the elderly and disabled has received significant attention from both industry and academia. The intelligent wheelchair/bed, which integrates the functionalities of a multifunctional nursing bed and an intelligent electric wheelchair, has become an important area of research in assistive robotics for the elderly and disabled. High-pre docking between the bed and wheelchair is a key challenge in the development of intelligent wheelchair/beds. Wheelchair/bed autonomous docking methods have evolved from magnetic strip navigation, machine vision combined with color tape navigation, machine vision with quick-response (QR) code navigation (Chen et al., 2018), light detection and ranging (LIDAR) navigation capable of autonomous path planning (Jiang et al., 2021), and multi-sensor fusion (Mishra et al., 2020). In the existing literature, pressure sensors have been employed for docking the wheelchair and bed (Mascaro and Asada, 1998), whereas other studies (Pasteau et al., 2016; Chavez-Romero et al., 2016; Li et al., 2019) have used machine vision to dock the wheelchair and bed, and some (Ning et al., 2017; Xu et al., 2022) have utilized LIDAR for wheelchair/bed autonomous docking. North Carolina State University designed a multi-sensor fusion simultaneous localization and mapping (SLAM) method based on depth cameras and LIDAR (Juneja et al., 2019). Shanghai University of Science and Technology introduced graph optimization algorithms for positioning in unknown environments, combining odometry and inertial measurement unit (IMU) data through extended Kalman filtering (EKF) for accurate autonomous indoor docking of intelligent wheelchair/beds (Su et al., 2021). However, the docking gap remains above 6 mm, requiring further improvements.

This study proposes an intelligent wheelchair/bed docking architecture based on multi-source information measurement. First, the mobile chassis of an intelligent robotic wheelchair/bed was improved with an independent four-wheel steering system, which structurally facilitates real-time omnidirectional corrections of errors detected during the docking process. Second, the traditional A* algorithm was improved to avoid diagonal crossings of obstacle vertices encountered during navigation. During the remote guidance phase, a laser radar and IMU fusion navigation system fixed on the wheelchair determined the wheelchair’s pose in the world coordinate system. This compensated for the onboard camera’s inability to directly observe the docking target, thus enabling visual guidance. In the medium-range navigation stage, a camera vision + IMU fixed on the wheelchair facilitated attitude positioning. At the close-range docking stage, a camera attached to the wheelchair enabled horizontal observation of landmark features on the auxiliary bed to determine the relative position and orientation between the wheelchair and the bed, thus enabling close-range visual docking.

## 2 Chassis design

**FIGURE 1:**
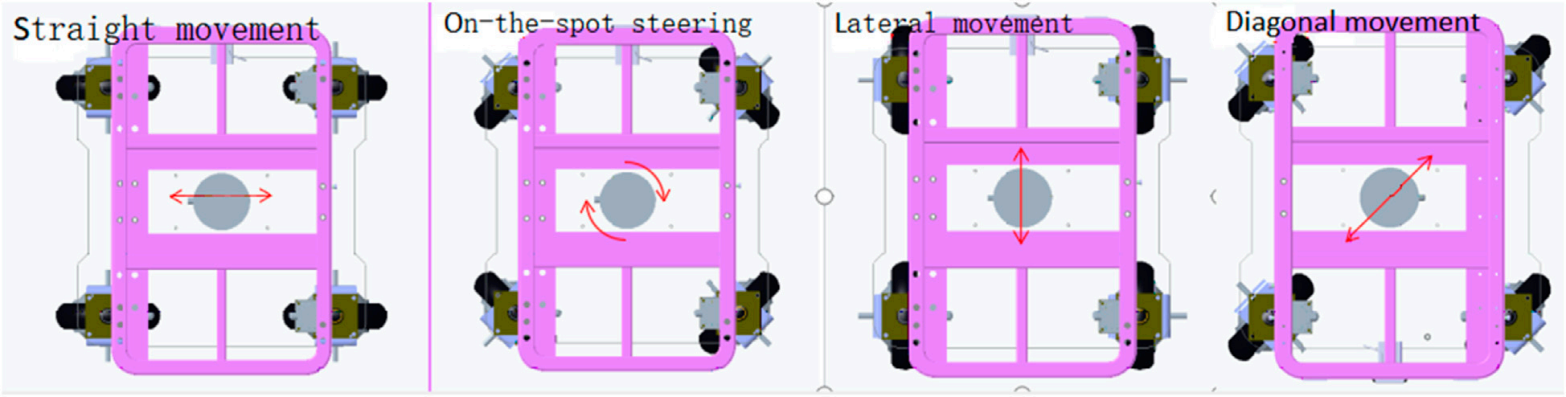
Improved motion structure of the wheelchair/bed chassis.

To meet the high-precision docking requirements of intelligent robotic wheelchair/beds, the mobile chassis must support on-the-spot steering to enable free maneuvering in narrow spaces, such as confined indoor home environments. The traditional wheel system of two active wheels plus two omni wheels has a large turning radius and lacks lateral movement capability, rendering it insufficient for high-precision docking of intelligent robotic wheelchair/beds. Therefore, the chassis of an intelligent robotic wheelchair/bed was upgraded to a four-wheel omnidirectional drive system, with four independently steered drive motors mounted in front and rear positions. Each drive motor is independently powered and controlled, enabling each wheelset to move independently. Figure 1 illustrates this structure.

Using this four-wheel independent drive steering configuration, the mobile chassis can perform straight, lateral, and diagonal movements, as well as on-the-spot steering, whereby the pose of the wheelchair/bed can be adjusted and corrected arbitrarily.

## 3 Proposed intelligent wheelchair/bed docking system

**FIGURE 2:**
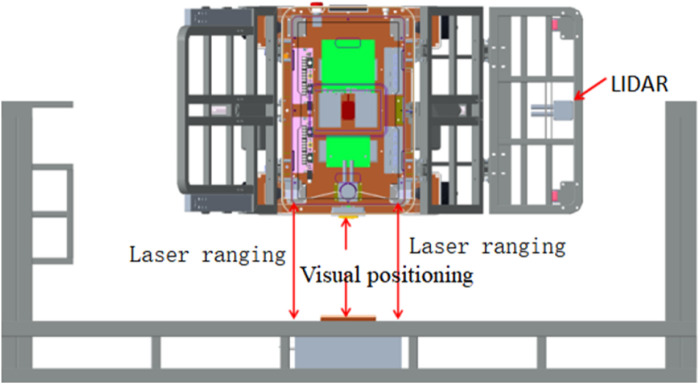
Schematic diagram of the intelligent wheelchair/bed docking system.

**FIGURE 3:**
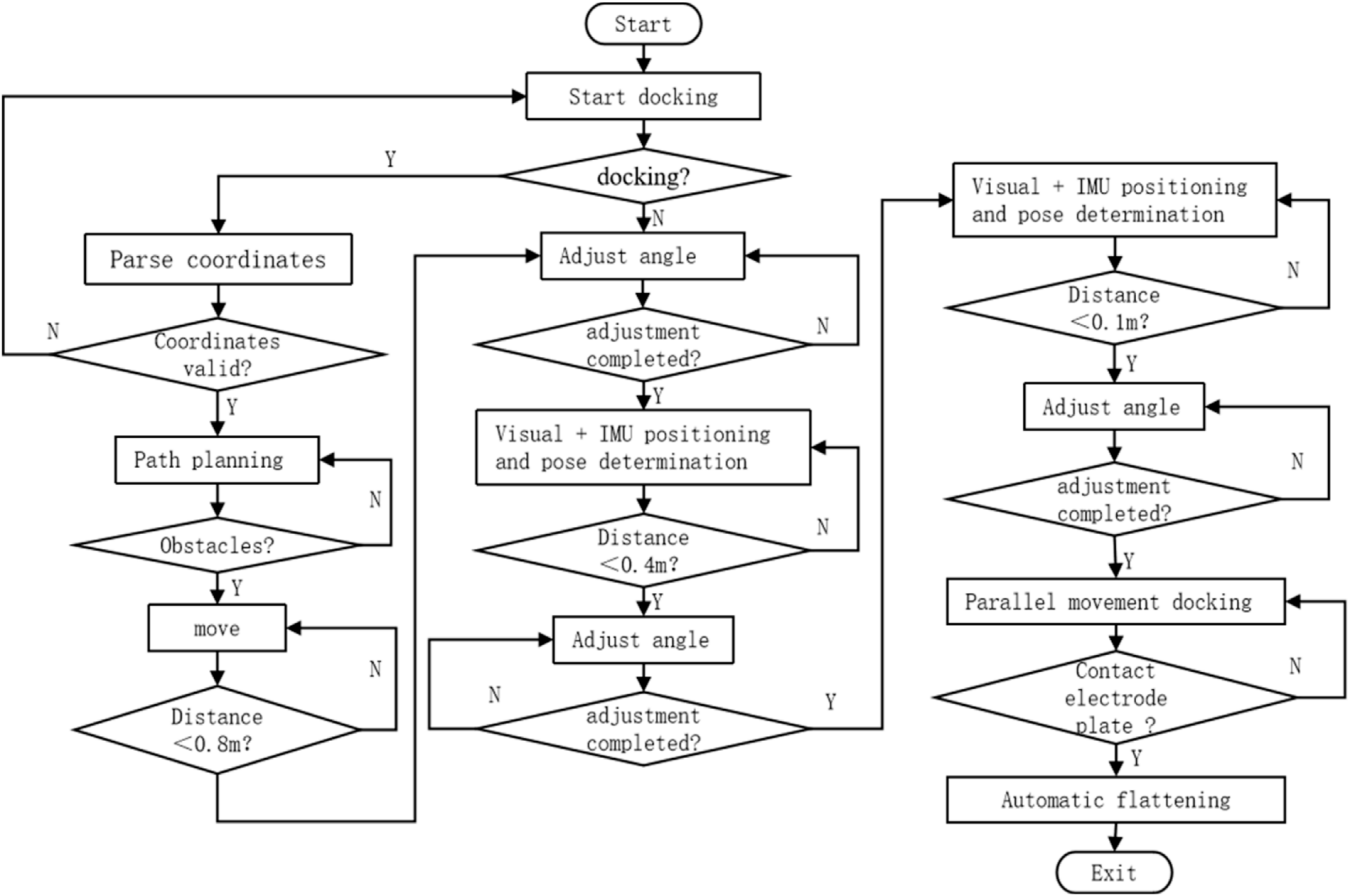
Flowchart of the intelligent wheelchair/bed docking process.

The intelligent wheelchair/bed docking system works in three phases: remote guidance, mid-range pose determination and positioning, and short-range precise docking. The schematic diagram and flowchart of the docking process are shown in Figures 2, 3, respectively.

1) Remote Guidance Phase: When the distance between the intelligent electric wheelchair and fixed bed frame exceeds 80 cm, pressing the “one-click recall” button on the bed frame initiates the remote guidance through path planning. The LIDAR, WLR-716 from Vanjee Technology, is mounted at the bottom of the footrest of the intelligent electric wheelchair, and the IMU (GNW-SurPass-A100) operates on an industrial personal computer (IPC) with a main control panel, M67_I526L (6360U), executing the relevant algorithms.
2) Mid-Range Pose Determination and Positioning Phase: When the distance between the intelligent electric wheelchair and fixed bed frame is 10–80 cm, the docking process employs machine vision combined with IMU for positioning and pose determination. The camera on the intelligent wheelchair/bed has a resolution of 1920 mm × 1080 mm and a frame rate of 30 fps.
3) Short-Range Precise Docking Phase: When the distance between the intelligent electric wheelchair and fixed bed frame is less than 10 cm, the camera in the machine vision system is in a defocused state. Precise docking is achieved through the signal feedback between a laser rangefinder and contact electrodes. The laser rangefinder, TF40, has an accuracy of ± 2 mm, a distance resolution of 1 mm, and a repeatability of less than 2 mm.

## 4 Construction of control system

**FIGURE 4:**
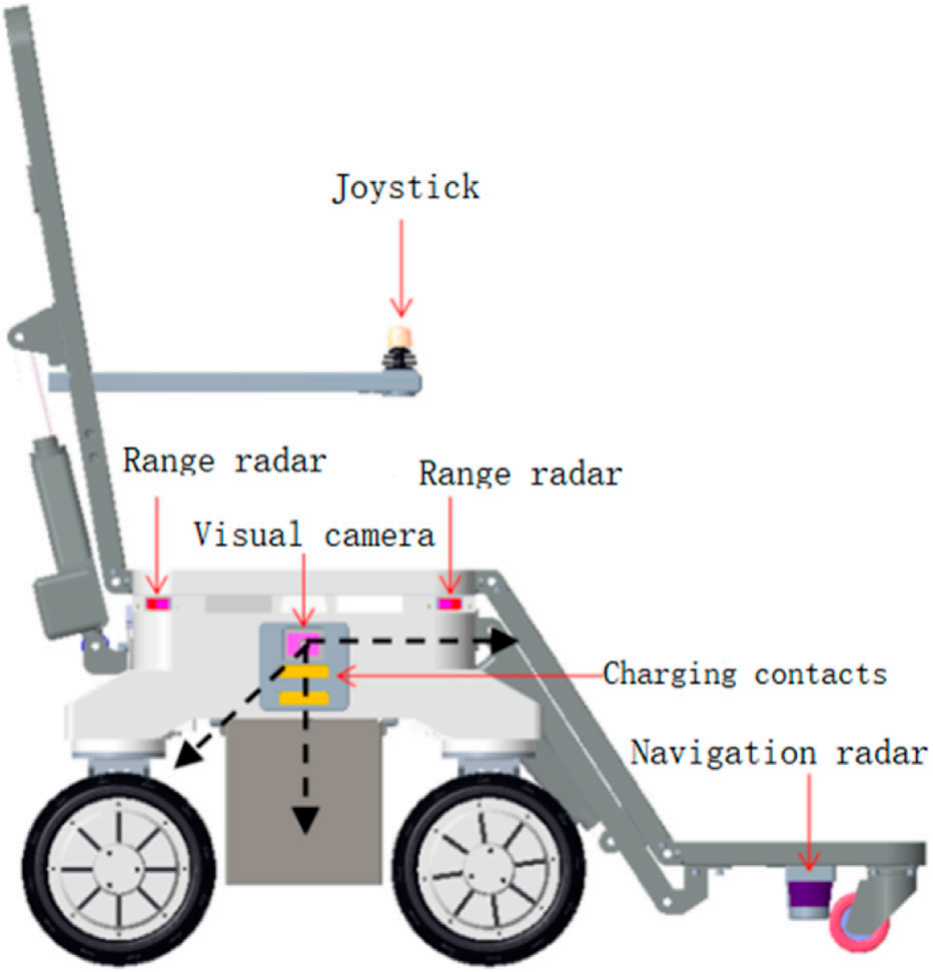
Camera, IMU, and intelligent electric wheelchair coordinate systems.

**FIGURE 5:**
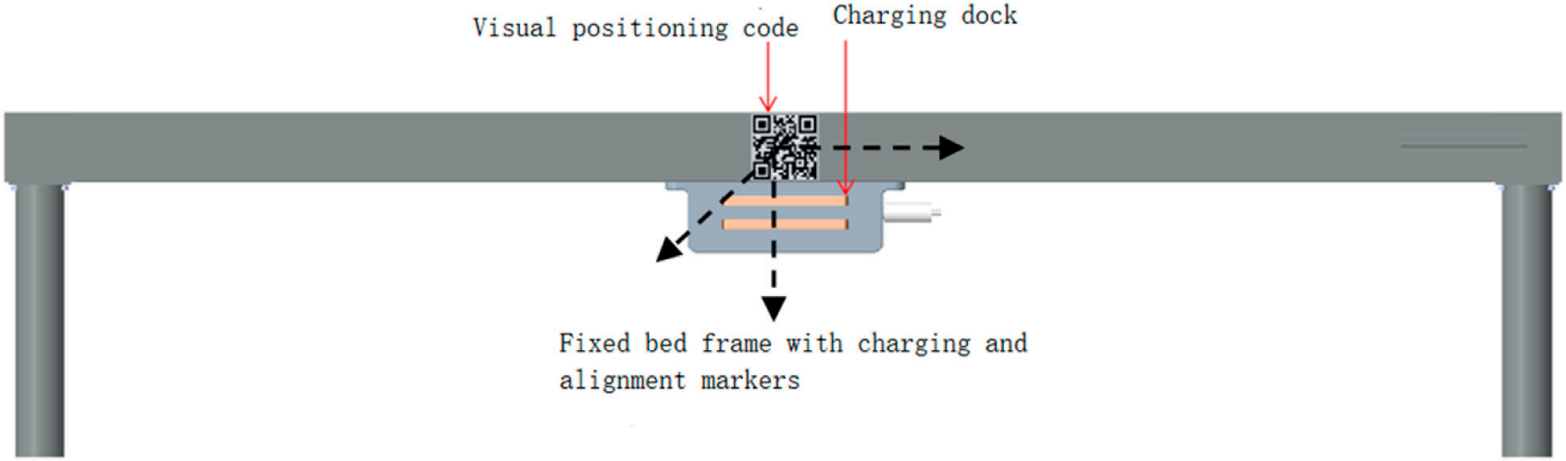
Fixed bed frame and world coordinate systems.

When the intelligent electric wheelchair, guided by LIDAR, is approximately 80 cm from the fixed bed frame, the camera mounted on the electric wheelchair (Figure 4) can clearly observe the QR code on the bed frame (Figure 5). Subsequently, the position and pose of the intelligent electric wheelchair are determined through visual and inertial fusion.

**FIGURE 6:**
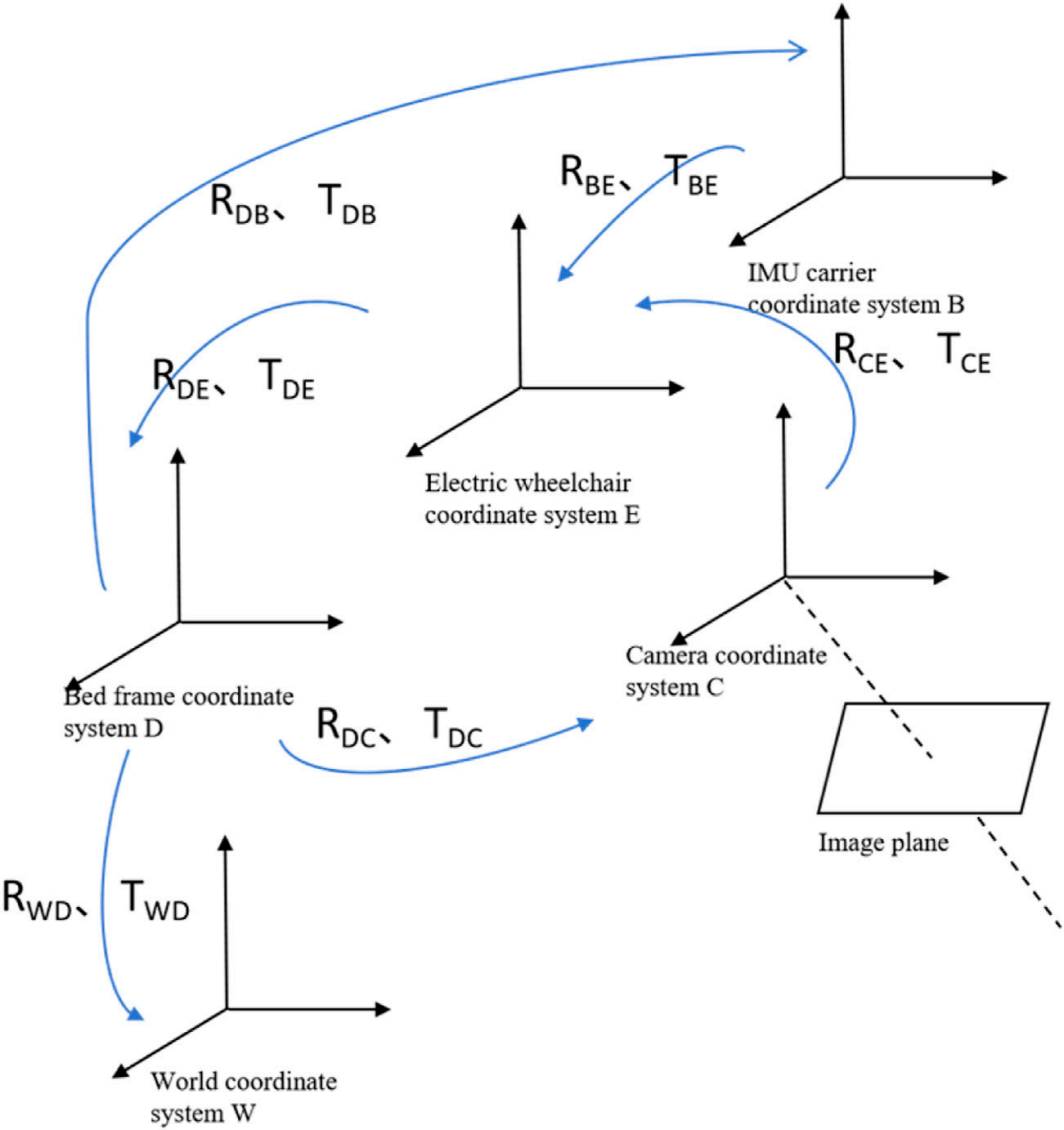
Visual-inertial positioning and pose determination coordinate system.

The coordinate systems of the camera, IMU carrier, intelligent electric wheelchair, fixed bed frame, and the world can be obtained through rotation and translation. The coordinate transformation relationships are shown in Figure 6. Setting the world coordinate system at the origin of the fixed bed frame coordinate system, the transformation matrix R_WD_ and translation matrix T_WD_ can convert the data from the bed frame coordinate system D to the world coordinate system W. Similarly, using the transformation matrices R_DE_, R_EB_, R_BC_, and translation matrices T_DE_, T_EB_, T_BC_, the data can be converted among their respective coordinate systems. The coordinate systems lie on the same horizontal plane; hence, 3D coordinates can be converted to 2D coordinates. External parameters are obtained by optimizing the Levenberg–Marquardt algorithm to obtain the transformation matrix between the fixed bed frame and camera coordinate systems.

Based on Equations 1, 2, the relative pose relationships between the electric wheelchair, IMU carrier, and world coordinate systems O_W_-X_W_Y_W_Z_W_ can be determined:

$$\left\{\begin{array}{l} R_{\text{WE}}=R_{\text{WD}}{\left(R_{\text{DE}}R_{\text{CE}}\right)}^{-1}\\ T_{\text{WE}=}=-T_{\text{WD}}{\left(T_{\text{DE}}T_{\text{CE}}\right)}^{-1}\left(R_{\text{DE}}T_{\text{CE}}+T_{\text{DE}}\right) \end{array}\right.$$ (1)

$$\left\{\begin{array}{l} R_{\text{WE}}=R_{\text{WD}}{\left(R_{\text{DE}}R_{\text{BE}}\right)}^{-1}\\ T_{\text{WE}=}=-T_{\text{WD}}{\left(T_{\text{DE}}T_{\text{BE}}\right)}^{-1}\left(R_{\text{DE}}T_{\text{BE}}+T_{\text{DE}}\right) \end{array}\right.$$ (2)

After obtaining the rotation matrix R and translation matrix T, the pose of the electric wheelchair in the world coordinate system O_W_-X_W_Y_W_Z_W_ is (*x*_*w*_, *z*_*w*_, *θ*_*w*_).

## 5 High-precision docking algorithm

### 5.1 Remote guidance algorithm

**FIGURE 7:**
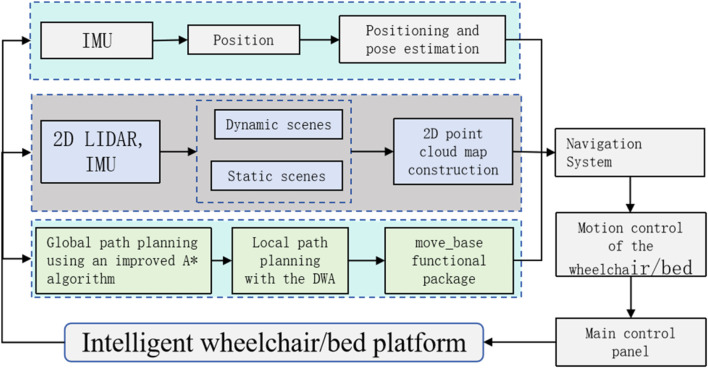
Block diagram of the intelligent wheelchair/bed remote guidance system.

The intelligent wheelchair/bed employs LIDAR and IMU fusion navigation for remote guidance. The system framework is shown in Figure 7.

As a classic heuristic graph search algorithm (Yue et al., 2021), the A* algorithm can be considered an extension of Dijkstra’s algorithm. It is one of the most effective algorithms in static global path planning (Dong et al., 2023; Zhang et al., 2020). The algorithm has been widely used in various applications. However, the traditional A* algorithm has some limitations, such as neglecting the robot’s shape and size, generating paths that may be too close to obstacles, and creating unsmooth paths composed of multiple discrete points.

**FIGURE 8:**
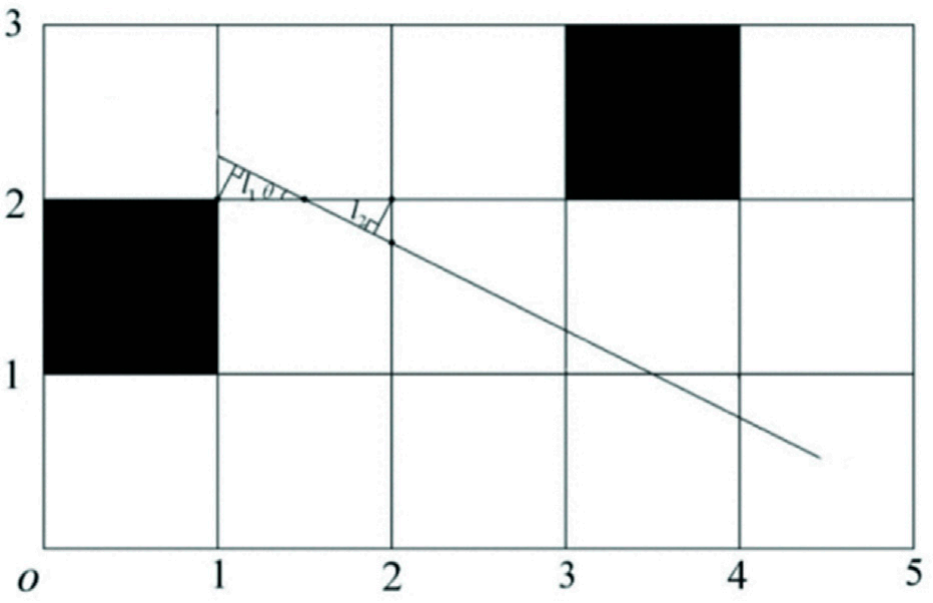
Optimal path planning method.

To address these limitations, the path equation was set as $y=k_{p}\left(x-x_{0}\right)+y_{0}$ , which determines whether the distance between the path and obstacle is safe. Here, the slope $k_{p}$ can be obtained from the coordinates of the starting point; $x_{0}$ and $y_{0}$ are the coordinates of the center of the adjacent obstacle on the left side of the line; $l_{1}$ and $l_{2}$ are the safety distances; and θ is the angle between the lines, ranging from 0° to 90°, as shown in Figure 8.

By continuously searching surrounding nodes and updating the optimal function, the shortest path can be obtained. The best solution is evaluated using the path cost estimation function (Mishra et al., 2020):

$$f\left(n\right)=g\left(n\right)+h\left(n\right)$$ (3)

In Equation 3, the *g(n)* is the estimated cost from the starting point to node *n*, and *h(n)* is the heuristic function, representing the estimated optimal path cost from node *n* to the target point.

Subsequently, a weighting factor γ (γ>1) is introduced to optimize the cost estimation function, as shown in Equation 4.

$$f\left(n\right)=g\left(n\right)+\gamma h\left(n\right)$$ (4)

Introducing the weighting factor γ increases the weight of the unknown path in the total path cost, thereby enhancing the search depth of the algorithm and facilitating the solution of the optimal path.

### 5.2 Mid-range pose determination and positioning algorithm

#### 5.2.1 Visual model

This study used a monocular high-speed camera to collect QR code information. Under the pinhole model projection, the 3D points are projected onto the normalized plane, yielding the coordinates of the 3D points on the image plane:

$$\left\{\begin{array}{c} u=f_{x}\frac{x}{z}+C_{x}\\ v=f_{y}\frac{y}{z}+C_{y} \end{array}\right.$$ (5)

Using homogeneous coordinates, Equation 5 can be transformed into Equation 6

$$Z\left(\begin{array}{c} u\\ v\\ 1 \end{array}\right)=\left(\begin{array}{ccc} f_{x} & 0 & C_{x}\\ 0 & f_{y} & C_{y}\\ 0 & 0 & 1 \end{array}\right)\left(\begin{array}{c} X\\ Y\\ Z \end{array}\right)≝KP$$ (6)

where *K* is a matrix of the camera’s intrinsic parameters; *f*_*x*_ and *f*_*y*_ are the scaling factors for the *x* and *y*-axes in the imaging plane and pixel coordinate system, respectively (pixel); *C*_*x*_ and *C*_*y*_ are the translation amounts (pixel); [*x*, *y*, *z*]^T^ are the coordinates of point *P* in 3D space.

The manufacturing errors in lenses and cameras may cause the collected image to be distorted radially and tangentially during translation and scaling. Therefore, Equation 7 is used to correct these distortions on the normalized plane.

$$\left\{\begin{array}{c} x_{correct}=x\left(1+k_{1}r^{2}+k_{2}r^{4}+k_{3}r^{6}\right)+2p_{1}xy+p_{2}\left(r^{2}+2x^{2}\right)\\ y_{correct}=y\left(1+k_{1}r^{2}+k_{2}r^{4}+k_{3}r^{6}\right)+2p_{2}xy+p_{1}\left(r^{2}+2y^{2}\right) \end{array}\right.$$ (7)

where, *k*_1_, *k*_2_ and *k*_3_ are the tangential distortion coefficients; *p*_1_ and *p*_2_ are the radial distortion coefficients; [*x*, *y*] and [*x*_*correct*_, *y*_*correct*_] are the coordinates before and after distortion correction, respectively; the square root of *r* ^*2*^ *= x* ^*2*^ *+y* ^*2*^ is the distance between the pixel and image center.

To obtain the pixel points on the pixel plane, the projection-corrected points need to be undistorted using the camera’s intrinsic parameters, as shown in Equation 8.

$$\left\{\begin{array}{c} u=f_{x}x_{correct}+C_{x}\\ v=f_{y}y_{correct}+C_{y} \end{array}\right.$$ (8)

#### 5.2.2 Inertial model

Inertial data are obtained from the IMU, and deterministic errors caused by biases, temperature, and scale errors can be excluded through prior calibration. Therefore, only random errors such as Gaussian white noise and biased random walk error need to be considered between the IMU and world coordinate systems. The IMU’s acceleration and angular velocity are modeled as shown in Equation 9.

$$\left\{\begin{array}{l} {\hat{\omega }}^{t}=\omega^{t}+b_{\omega t}+\eta_{\omega }\\ {\hat{a}}^{t}=a^{t}+b_{at}+\eta_{a}+R_{bw}g^{w} \end{array}\right.$$ (9)

where ω and a are the measured angular velocity and acceleration, respectively; $\hat{\omega }$ and $\hat{a}$ are the observed values; $b_{\omega t}$ and $b_{at}$ are the angular velocity and acceleration biases, respectively; $\eta_{\omega }$ and $\eta_{a}$ are the measurement noises; $R_{bw}$ is the transformation matrix from IMU to the world coordinate system; $g^{w}$ is the gravity in the world coordinate system.

From Equation 9, the kinematic equation of the intelligent wheelchair/bed can be obtained as follows:

p˙wbt=vtwv˙tw=awbt=△pwot△t2q˙wbt=qwbt⊗012ωbt (10)

where $p_{{wo}_{t}}$ is the displacement increment obtained by IMU, and ⊗ denotes quaternion multiplication. Based on the state of the intelligent wheelchair/bed at time *i* and Equation 10, ignoring biases and noise, the state at time *i*+1 can be obtained as shown in Equation 11:

$$\left\{\begin{array}{l} p_{{wb}_{i+1}}=p_{{wb}_{i}}+v_{i}^{w}△t+R_{{wb}_{i}}\alpha_{b_{i}b_{i+1}}\\ v_{i+1}^{w}=v_{i}^{w}+R_{{wb}_{i}}\beta_{b_{i}b_{i+1}}\\ q_{{wb}_{i+1}}=q_{{wb}_{i}}⊗q_{b_{i}b_{i+1}} \end{array}\right.$$ (11)

the α, β, γ are shown in Equation 12 where

$$\left\{\begin{array}{l} \alpha_{b_{i}b_{i+1}}=\frac{1}{2}\left[q_{i}\left({\hat{a}}_{i}-b_{ai}\right)+q_{i+1}\left({\hat{a}}_{i+1}-b_{ai}\right)-2g^{w}\right]\Delta t^{2}\\ \beta_{b_{i}b_{i+1}}=\left[q_{i}\left({\hat{a}}_{i}-b_{ai}\right)+q_{i+1}\left({\hat{a}}_{i+1}-b_{ai}\right)-2g^{w}\right]\Delta t\\ q_{b_{i}b_{i+1}}=\frac{1}{2}\left({\hat{\omega }}_{i}+{\hat{\omega }}_{i+1}\right)-b_{\omega i} \end{array}\right.$$ (12)

$Z_{b_{i}b_{j}}=\left[\alpha_{b_{i}b_{i+1}},\beta_{b_{i}b_{i+1}},q_{b_{i}b_{i+1}}\right]$ is the pre-integrated measurement value of the IMU. It is treated as a constant during the continuous motion of the intelligent wheelchair/bed.

#### 5.2.3 Visual/IMU positioning and pose determination algorithm

**FIGURE 9:**
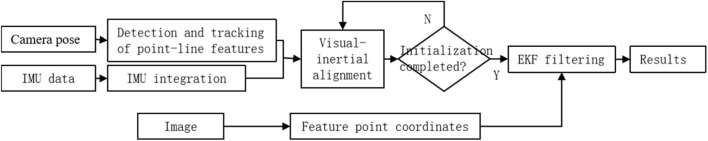
Flowchart of visual and IMU positioning and pose determination.

The algorithms proposed by Niu et al. (2023) and Li et al. (2023) can achieve the expected positioning accuracy, meeting the precision requirements for the mid-range positioning and pose determination of the intelligent wheelchair/bed. Inspired by this, a tightly coupled visual/IMU algorithm was adopted for the mid-range docking of the intelligent wheelchair/bed. The algorithm framework is illustrated in Figure 9. First, initialization is performed to completely calibrate IMU drift, gravity vector, and external parameters from the camera to the IMU; this ensures alignment of the sensors. Subsequently, the camera detects changes in the coordinates of the target in the system to obtain pose information. The obtained pose information is compared with the desired pose information, driving the intelligent wheelchair/bed’s motion system toward the fixed bed frame. Mid-range positioning and pose determination are terminated when the visual sensor is out of focus.

### 5.3 Short-range precise docking algorithm

When the selected visual sensor is out of focus at a distance of 10 cm from the fixed bed frame, two laser-ranging sensors mounted on the intelligent wheelchair/bed are used to measure the distance between the intelligent wheelchair/bed and fixed bed frame. Thus, the tilt angle between the intelligent wheelchair/bed and fixed bed frame is calculated, achieving short-range precise docking.

**FIGURE 10:**
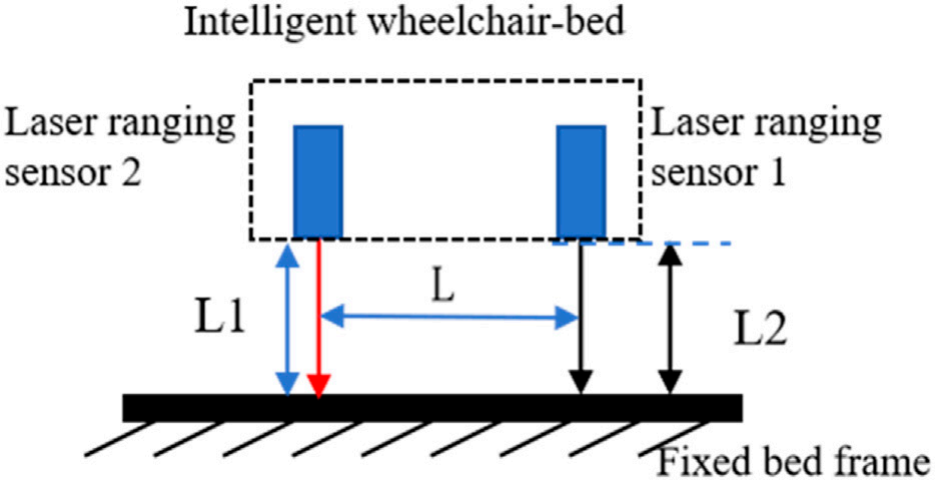
Laser triangulation ranging system model.

As shown in Figure 10, the laser triangulation ranging system consists of two laser rangefinders mounted on the intelligent wheelchair/bed and fixed bed frame. The distance between the laser rangefinders is L; the distance between laser rangefinder 1 and fixed bed frame is L1; and the distance between laser rangefinder 2 and fixed bed frame is L2. The tilt angle between the intelligent wheelchair/bed and fixed bed frame is calculated using Equation 13.

$$\tan\propto =\frac{\left|L1-L2\right|}{L}$$ (13)

Based on the tilt angle and distance, the four-wheel drive chassis adjusts the intelligent wheelchair/bed’s tilt angle, longitudinal offset, and lateral offset relative to the fixed bed frame. The automatic docking stops when the docking feedback signal is received.

## 6 Experimental results and analysis

**FIGURE 11:**
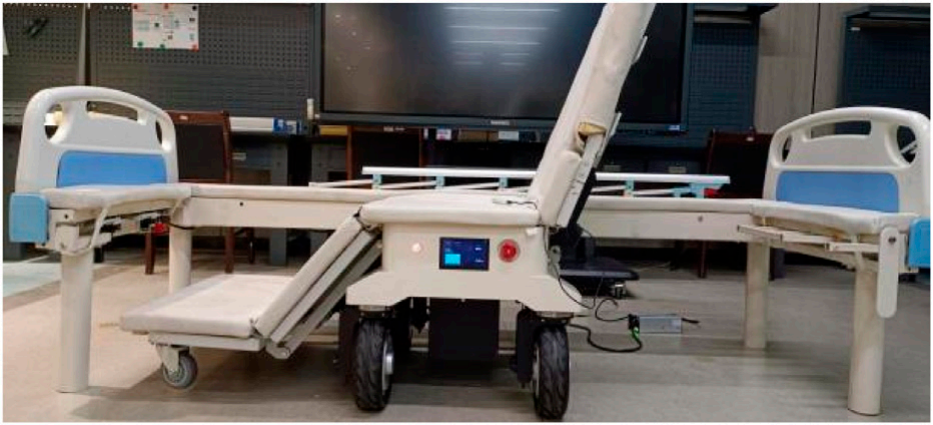
Physical platform for validating the docking of the intelligent wheelchair/bed.

To verify the effectiveness of the proposed method, autonomous docking experiments were conducted on a self-developed intelligent wheelchair/bed prototype (Figure 11). A coordinate system was established with the fixed bed frame as a reference. The results of docking from different starting points of the mobile part of the intelligent wheelchair/bed are presented in Table 1. The initial pose in Table 1 was measured manually, and the final pose was the pose after the intelligent wheelchair/bed completed docking and automatically charged.

**TABLE 1:** Docking errors of the intelligent wheelchair/bed from different starting points.

Initial pose/m	Average docking gap/mm			Docking angle deviation/°		
( $x_{w}^{0}$ , $z_{w}^{0}$ ,θ^0^/(°))	This paper	Reference 7	Reference17	This paper	Reference7	Reference17
(3.0,12.0,90)	2.8	6.2	6.1	0.2	0.4	0.4
(-3.0,12.0,90)	3.0	6.1	6.2	0.2	0.4	0.4
(1.3,4.0,60)	3.5	6.5	7.8	0.2	0.4	0.5
(1.3,4.0,-60)	3.2	6.7	7.7	0.2	0.4	0.5
(-1.3,4.0,60)	3.2	6.7	7.7	0.2	0.4	0.5
(0.7,0.8,15)	2.4	4.2	7.6	0.2	0.3	0.5
(0.7,0.8,-15)	2.6	4.0	7.4	0.2	0.3	0.5
(-0.7,0.8,15)	2.6	4.2	7.4	0.2	0.3	0.5

Note: The average docking error is measured at the center position of the fixed bed frame, and the data in the table is the average of 10 consecutive measurements.

Table 1 indicates that in the final position, the proposed three-phase docking method with multi-sensor fusion achieves an average docking gap error of less than 5 mm and a docking angle deviation of less than 0.5°, outperforming the methods proposed by Li et al. (2019) and Fang et al. (2022). The proposed method can guide the mobile part of the intelligent wheelchair/bed to the specified position with high precision.

## 7 Conclusion

(1) A mobile chassis with independent four-wheel drive and steering capabilities was designed to structurally solve the free movement problem of the wheelchair/bed, thereby offering a mechanical structure that guarantees high-precision docking.
(2) A three-phase docking method integrating multi-source information was proposed to enable the high-precision docking of the wheelchair/bed. In the remote guidance phase, a docking approach combining LIDAR/IMU and an improved A* algorithm was employed to achieve indoor positioning, navigation, and obstacle avoidance of the wheelchair. During the mid-range pose determination and positioning phase, a visual/IMU was used for guiding the intelligent wheelchair/bed to a distance of 100 mm from the bed frame. When the distance between the wheelchair/bed and the bed frame was less than 100 mm, laser triangulation ranging was employed to achieve autonomous docking between the wheelchair and the bed.
(3) Practical prototype testing revealed that the designed three-stage docking method improved the docking accuracy of the intelligent wheelchair/bed to within 4 mm. This represents a significant improvement compared to the methods described by Li et al. (2019) and Fang et al. (2022), thereby verifying the feasibility of the proposed method.
(4) To achieve safe autonomous movement and high-precision docking of intelligent wheelchair/beds in unstructured indoor dynamic environments, future research will focus on key technologies such as autonomous positioning and semantic mapping, as well as collision-free automatic high-precision docking control between the wheelchair and bed frame or bucket.

## Data Availability

The original contributions presented in the study are included in the article/supplementary material, further inquiries can be directed to the corresponding author.
